# Adult Onset Sacrococcygeal Teratoma

**DOI:** 10.7759/cureus.45291

**Published:** 2023-09-15

**Authors:** Shathak S Baikady, Nagesh K Singaram

**Affiliations:** 1 Surgical Oncology, Sri Venkateswara Institute of Medical Sciences, Tirupathi, IND; 2 General Surgery, Vydehi Institute of Medical Sciences and Research Centre, Bangalore, IND; 3 Surgical Oncology, Sri Venkateswara Institute of Medical Sciences, Tirupati, IND

**Keywords:** surgical excision, benign, adult onset, sacrococcygeal, teratoma

## Abstract

Sacrococcygeal teratoma (SCT), one of the most common neoplastic tumors in newborns, is found very rarely in adults. These teratomas are germ cell tumours. Most of these tumors are benign and cystic in nature, with only 1-2% of them having a malignant transformation. Most of these tumors are benign and cystic in nature, with only 1-2% of them having malignant transformation. A higher incidence was seen in females. Typically, cystic teratomas are asymptomatic, and so the diagnosis was often made inadvertently during radiographic studies. The majority of treatment is complete surgical excision, and both open and laparoscopic procedures have been proven to be efficient. Histopathologic examination can confirm the diagnosis. We present this unusual instance of a 56-year-old female patient with a sacrococcygeal teratoma.

## Introduction

Teratomas are very rare tumors that develop from pluripotent embryonic stem cells. Sacrococcygeal teratomas (SCT) are tumors that include tissues generated from two or more primitive germ cells and are the most prevalent extragonadal germ cell tumors [1]. With a prevalence rate of one in 40,000 and a female-to-male ratio of 4:1, they are the most common fetal neoplasms. Fetal ultrasonography can be used to make a prenatal diagnosis, and 50-70% of them are discovered within the first few days of life [2]. SCT is extremely rare in adults, with only a few cases found in literature, and among them, prevalence predominates three to four times more often in adult females than in adult males [1]. Rarely, these adult tumors will undergo malignant transformation, and there is also an infection risk of 20-30% as they are typically benign cystic tumors [3]. Altman divided SCTs into four categories based on their location: type I, which is primarily external with a small presacral component; type II, which is external with an intrapelvic component; type III, which is external with a pelvic and abdominal component; and type IV, which is internal with an intrapelvic and abdominal location. Types II and III are frequently dumbbell-shaped [1]. The teratoma's mass influences nearby organs such as the rectum, urinary bladder, and pelvic vasculature, which results in obstructive symptoms of the affected system. This is the primary cause of the clinical manifestations of the teratoma. Along with severe skin excoriation, ulceration, and deformity, the mass's external projection will also be uncomfortable and disfiguring [4].

When it comes to diagnosing these lesions and determining their origin, extent, and relationship to the abdominal and pelvic organs, CT scans and/or MRI imaging are invaluable tools. When the coccyx is removed along with the lump and the histological report demonstrates that the teratoma is benign, surgical excision is regarded as curative [5]. The cysts may be bordered by genuine epithelium and contain serous fluid, mucoid, or sebaceous material [6]. Malignant SCTs have a bad prognosis; hence, early surgical resection is necessary. For tumor resection, transabdominal, trans-sacral, or a combination of both of the techniques are used. Recent instances of tumor laparoscopic excision of the tumor is used for transabdominal techniques [7]. To fill the gaps in literature about the SCTs in adults, especially in India, we present this unusual case of an adult sacrococcygeal teratoma that was treated in our hospital.

## Case presentation

**Figure 1:**
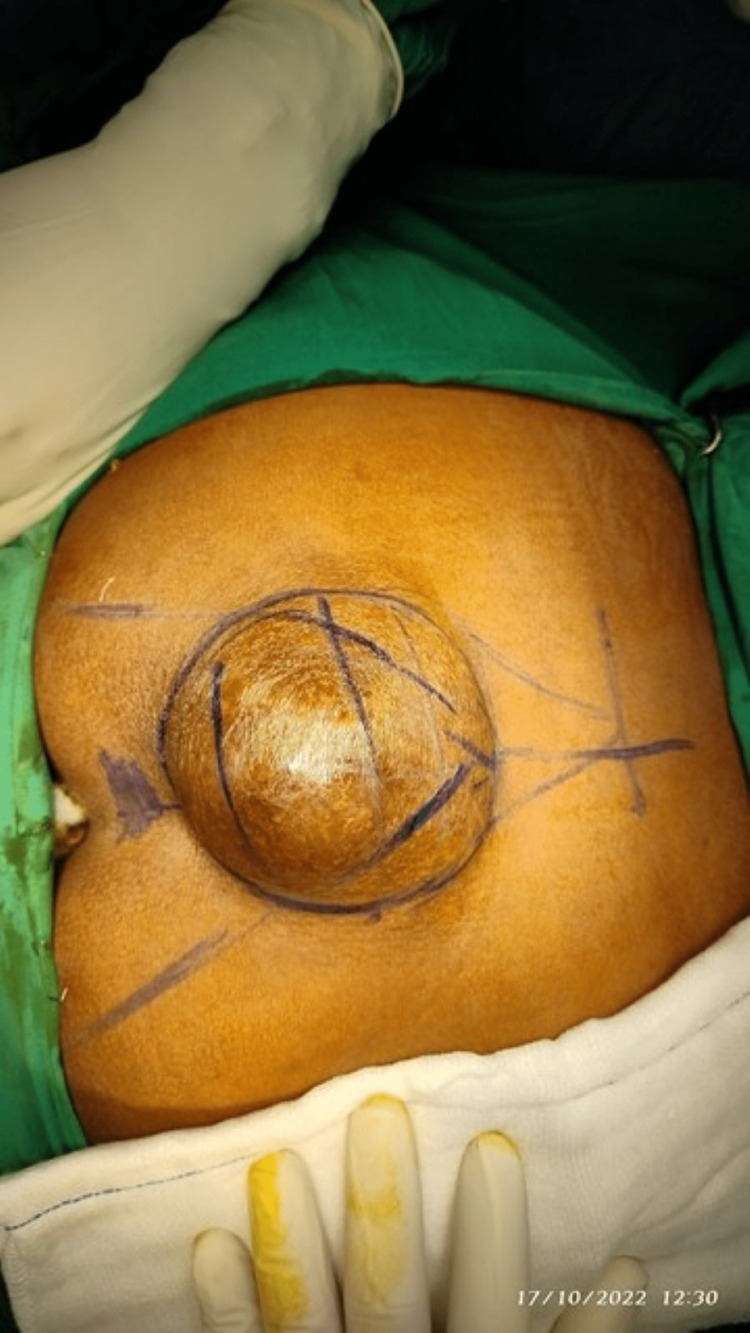
Clinical presentation of the swelling in the sacral region

A 56-year-old female patient presented to our outpatient department with swelling in the sacral region on the posterior aspect for the past seven months. Upon taking the history, she stated that the swelling had a gradual onset and has been progressively increasing in size. There were no associated symptoms. She had a history of similar complaints in the past and underwent surgery seven years ago. However, she couldn't recollect the details of that surgery and didn't have any reports for it. On examination, we identified a solitary swelling of approximately 9x5 cm was present over the sacrum (Figure 1). The borders of the swelling were well-defined, and a positive transillumination test was observed. We could not arrive at a diagnosis based on the history and examination, so MRI was advised.

**Figure 2:**
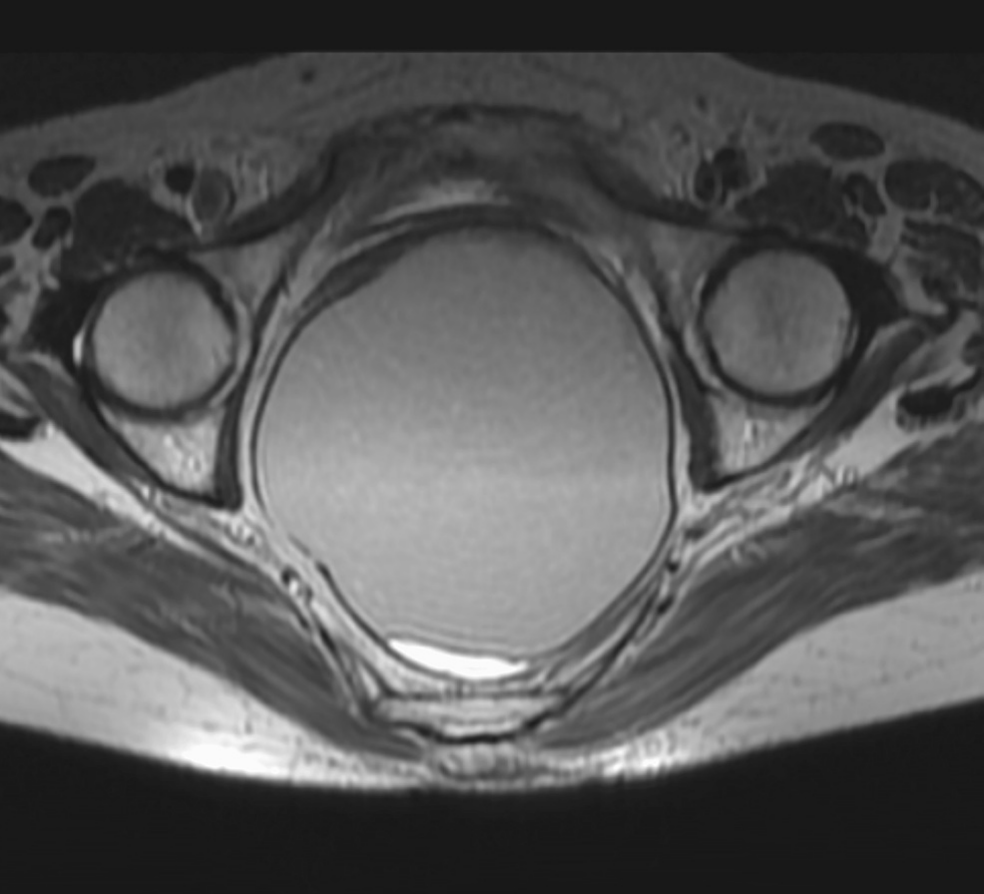
MRI of the swelling revealing a cystic lesion

MRI report showed a large cystic lesion with intra and extra pelvic components, measuring approximately 12x8.4x12.3 cm, and is located in the midline. The extra pelvic component was also seen extending up to skin and subcutaneous tissue. There was no solid component or fat in the lesion. The mass was seen compressing and displacing the rectum and uterus anteriorly. There was no bony erosion and no involvement of spinal nerve roots (Figure 2). These findings were suggestive of either a mature cystic teratoma or a cystic lymphangioma. Therefore, surgical excision was planned for the patient.

**Figure 3:**
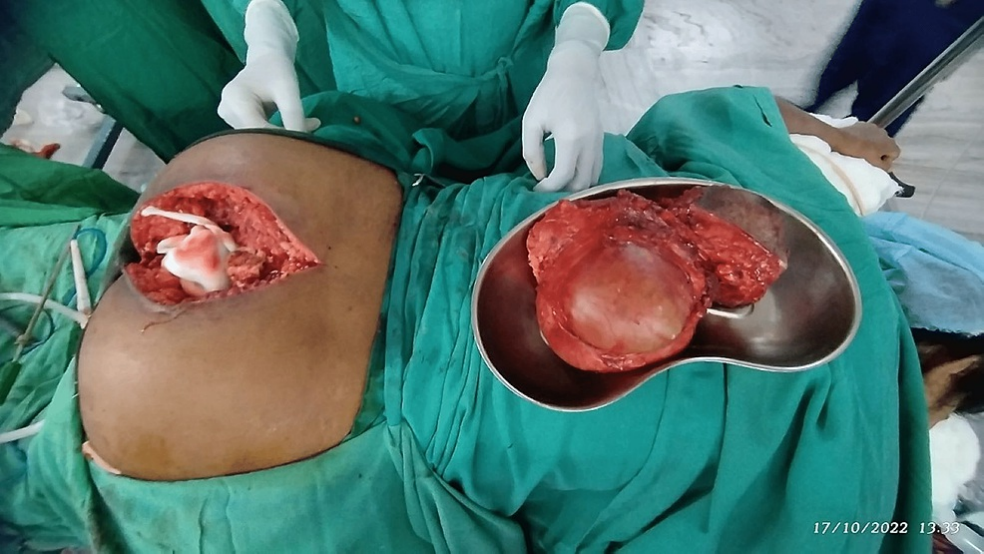
Excision of the intra- and extra-pelvic components of the lesion

The 15x10x12 cm intrapelvic cystic lesion, along with a 7x5x6 cm lesion located posterior to the coccyx in the subcutaneous plane, were identified first. The pelvic component of the cystic lesion was identified and carefully dissected from the surrounding peritoneum. Additionally, a total abdominal hysterectomy with bilateral salpingo-oopherectomy was performed. The bladder was reflected anteriorly, and the rectum was positioned to the anterior aspect. The patient was shifted to the prone position, and a lower vertical midline incision was made around the cystic lesion, securing it over the sacrum. So, a combined transabdominal and sacral approach was used to take out the lesion. The subcutaneous tissue and muscles were separated all around, and the rectum was identified and pushed laterally. The intra-pelvic and extra-pelvic components were completely removed (Figure 3).

The lesion was sent for histopathological examination. Multiple sections were studied from the cystic mass, showing the surface skin with keratinized stratified squamous epithelium and an unremarkable dermis. The subcutis has shown a multiloculated cyst lined by stratified squamous epithelium and pseudostratified ciliated columnar epithelium. The cyst wall showed mature adipose tissue, smooth muscle bundles, and foci of lymphoid aggregates. The cyst wall is surrounded by foreign body type of giant cells. The lumen of the cyst shows lamellated keratin. The microscopic examination revealed features consistent with cystic teratoma.

## Discussion

The present patient is a classic case of adult sacrococcygeal teratoma, presenting with swelling on the sacral region without any significant symptoms. MRI confirmed a cystic sacrococcygeal mass in the midline with intra- and extra-pelvic lesions. Teratomas are tumors made up of many cell types that come from multiple germ layers, and the coccyx is the most common extragonadal site [8]. In contrast to neonatal SCT, which typically manifests as an externally visible mass, adult SCT is typically observed as an intra-pelvic mass. The origins of SCT have been the subject of numerous theories, one of which involves multipotent cells originating in Henson's nodes and moving caudally to rest in the coccygeal area [9].

Adult patients with SCT may not exhibit any symptoms, and the diagnosis is made as a result of an unanticipated retro-rectal mass discovered during a digital rectal examination or other pertinent radiological studies. Occasionally, they may exhibit vague symptoms or symptoms brought on by the compression of pelvic viscera [10]. These symptoms can include mild, non-specific back discomfort, constipation, frequent urination, or dysmenorrhea. Our case was asymptomatic in nature, and there were no elevated tumor markers suggestive of malignancy [11]. The cornerstone of an investigative workup is an imaging study. Usually, heterogenic echogenicity with cystic and solid portions can be seen during an ultrasound examination of the cyst. Computerized tomography (CT) and magnetic resonance imaging (MRI) are now accepted as standards [12]. The mixed cystic-solid character of the mass is defined by the CT, but the MRI provides a superior tomographic evaluation and cyst evaluation, allowing for more effective pre-operation staging and planning. Since these tumours develop from germ cells, serum tumor markers such as alpha fetoprotein (AFP), human chorionic gonadotropin (hCG), and lactate dehydrogenase (LDH) should be checked, and any elevation could be an indication of a malignant change [13]. Fine needle aspiration cytology may be helpful in understanding the nature of the mass, but we had not performed it in our case. Chordoma, meningocele, giant cell tumor of the sacrum, osteomyelitis of the sacrum, pilonidal cysts, rectal duplication cysts, fistula with presacral extension and abscess formation, post-injection granuloma, and tuberculosis are among the differential diagnoses of SCT in adults [14]. The histopathological examination in the present case demonstrated the benign nature of the cyst as a mature teratoma.

The primary and most effective form of treatment for SCT is total surgical excision. It has been demonstrated that open and laparoscopic techniques are equally successful. The size, location, and components of the tumor determine the surgical strategy and surgical resection technique that should be used. The transabdominal route is frequently chosen for high lesions and offers excellent tumor posterior to the rectum exposure. The trans sacral technique is thought to be a promising alternative for tumors with a diameter of 10 cm or less and a location below the S3 vertebra [15]. In our case, due to the substantial extra-pelvic extension and doubtful adhesions to the rectum, we opted for the combined approach. The danger of recurrence (30-40%) means that excision of the coccyx may be required. This is because the bone may contain a nidus of pluripotent cells. Complete excision is sufficient for tumors with a benign histology [16]. Surgery alone is insufficient to remove malignant teratomas; patients also need adjuvant radiation and/or chemotherapy.

## Conclusions

Sacrococcygeal tumors are rare extra gonadal teratomas seen in adults, and they are much harder to diagnose due to the lack of associated symptoms. Diagnosis mainly relies on clinical examination and imaging. Therefore, a preoperative MRI of the pelvis might help in the appropriate planning of the surgical procedure and classification of SCT. The choice of surgical approach depends on the size, location, and components of the tumor. Surgical excision of the lesion is the preferred definitive modality of treatment for sacrococcygeal teratoma. Adults with mature sacrococcygeal teratomas rarely develop malignancies, and close follow-up is crucial for spotting early recurrence.
